# An Amplatzer Septal Occluder Trapped in the Left Ventricular Outflow Tract: A Case Report

**DOI:** 10.7759/cureus.73244

**Published:** 2024-11-07

**Authors:** Nicolas Bellofatto Piazza, Mohamed Ben Yedder, Marie Delmas, Badih El Nakadi

**Affiliations:** 1 General Surgery, Université Libre de Bruxelles, Brussels, BEL; 2 Cardiology, HUmani - CHU Charleroi-Chimay, Lodelinsart, BEL; 3 Anesthesiology, HUmani - CHU Charleroi-Chimay, Lodelinsart, BEL; 4 Cardiothoracic Surgery, HUmani - CHU Charleroi-Chimay, Lodelinsart, BEL

**Keywords:** amplatzer septal occluder, embolisation, inter-atrial defect, interventional cardiology, migration

## Abstract

We present the case of a 32-year-old female patient who presented at the cardiology consultation with shortness of breath and palpitations. A large inter-atrial defect was identified through echocardiography, prompting the cardiology team to perform a percutaneous closure procedure using the Amplatzer Atrial Septal Occluder (Abbott Laboratories, Abbott Park, IL, USA). Seven weeks later, a migration of the prosthetic device into the left ventricular outflow tract was diagnosed. A surgical procedure was immediately performed to explant the device and repair the defect. This case highlights the importance of vigilant monitoring in patients undergoing percutaneous closure procedures to detect severe complications such as device migration at an earlier stage.

## Introduction

Atrial septal defects (ASDs) are common congenital cardiac anomalies with a worldwide prevalence of 1.64/1000 live births and representing 15.37% of congenital heart diseases [1,2]. Among the various types of ASDs, ostium secundum ASDs are the most frequently encountered, accounting for approximately 75% of cases. A significant number of individuals remain undiagnosed until adulthood [3].

Untreated ASDs can lead to significant complications, including pulmonary hypertension, heart failure, arrhythmias, paradoxical embolism and infectious endocarditis [4]. Clinically, patients often present with reduced functional capacity and symptoms such as dyspnea and palpitations associated with supraventricular tachyarrhythmias. Less commonly, recurrent pulmonary infections and right heart failure may occur [4].

With advancements in interventional cardiology, percutaneous closure techniques using transcatheter atrial septal occluders (ASO) have gained popularity as a less invasive alternative compared to traditional surgical closure of ASDs [5]. ASO are designed as self-expandable nitinol wire mesh disks and have been associated with lower rates of total or major early post-procedural complications compared to open-heart surgical repair [5,6].

Up to 80% of ostium secundum ASDs, regardless of the defect size, are suitable for percutaneous closure with the Amplatzer ASO (Abbott Laboratories, Abbott Park, IL, USA) [7]. However, the morphology of the ASD rims is the primary limiting factor. Specifically, an absent or deficient rim toward the coronary sinus, inferior-posterior rim (toward the inferior vena cava), posterior rim (toward the pulmonary veins), superior-posterior (toward the superior vena cava), and inferior (toward the AV valves) are contraindications to percutaneous closure and require surgical closure [7]. The reported rate of adverse events linked to the Amplatzer ASO is 5.1%, including a rate of arrhythmias of 1.8%, standing out as the most prevalent complication. Device embolization, although relatively rare, occurs in 0.7% of cases [8].

Instances of device embolism have been documented across the four cardiac chambers, most often in the left cavities, as well as in the aorta and pulmonary arteries [9,10]. The causes of migration include inappropriate sizing, floppy interatrial septum, inaccurate deployment and tearing of the ASD during device implantation [9, 11]. Other potential predictors of ASO dislodgment include a pulmonary to systemic blood flow ratio (Qp/Qs) exceeding 3.13, inter-atrial septum erosion and aneurysm formation after implantation [12].

Device migration is predominantly identified during or shortly after the procedure but can also manifest very late, even years after implantation [9,13]. In such cases, percutaneous retrieval is successful in 16.7% of cases, while the remainder require operation. Device embolisation has a mortality rate of 1.8% [10].

We present a case of percutaneous ASD closure in an adult patient, along with the subsequent complication of device migration necessitating reoperation. We aim to highlight this rare occurrence and emphasize the importance of careful monitoring and management in such cases.

## Case presentation

**Figure 1:**
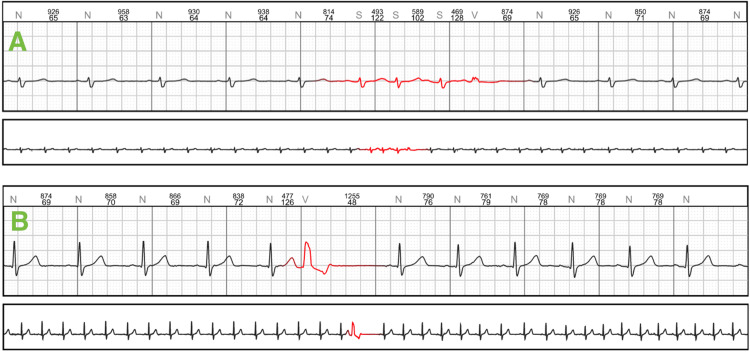
Preoperative Holter study A: triplet of supra ventricular extrasystoles B: ventricular extrasystole

A 32-year-old female patient presented to the cardiology consultation complaining of shortness of breath and palpitations for approximately two weeks. The patient had no significant medical history and initial laboratory tests were within normal limits. Seven-day Holter monitoring showed no atrial fibrillation, 59 ventricular and 19 supra ventricular extrasystoles (Figure 1).

**Figure 2:**
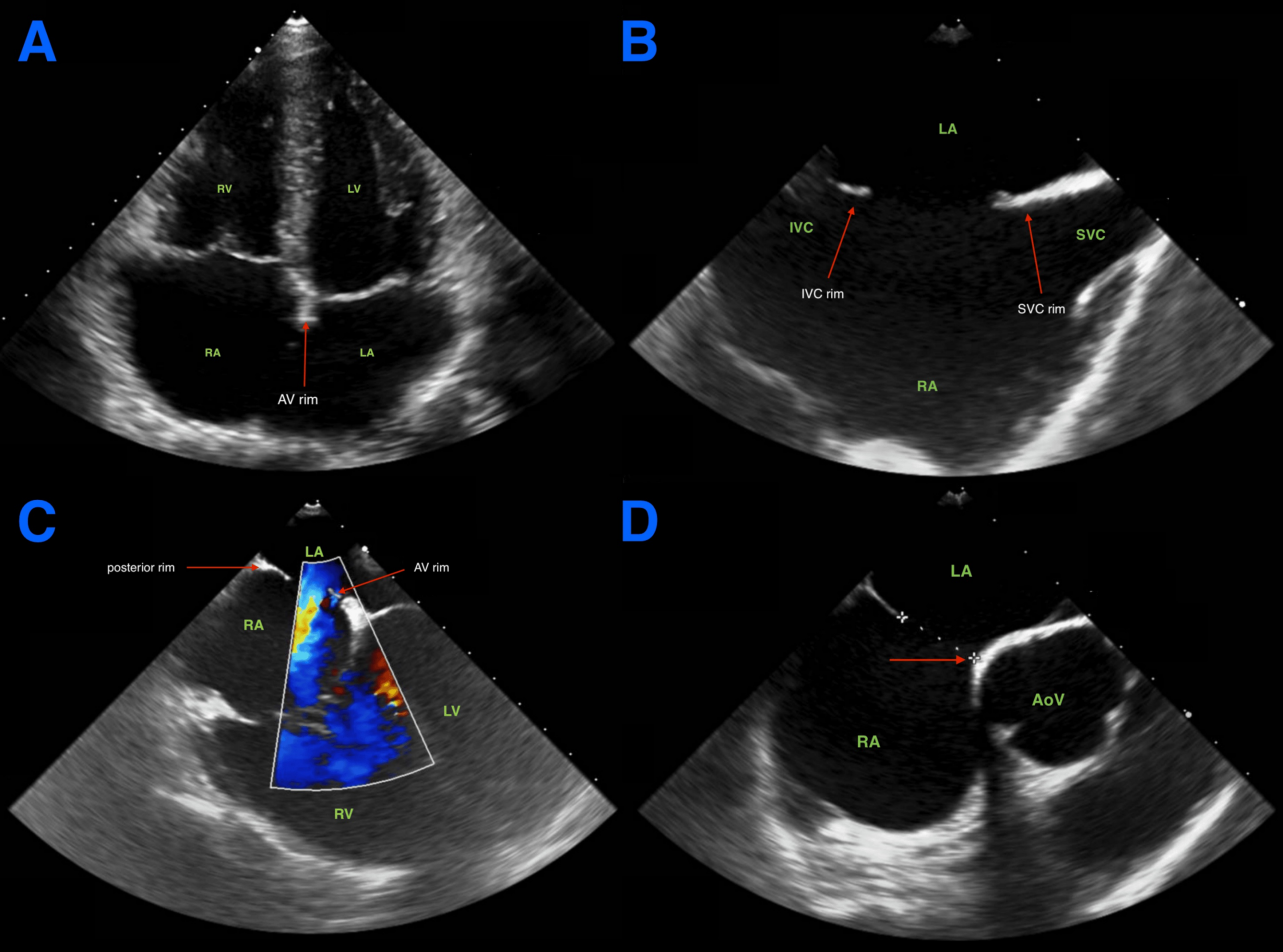
Preoperative echocardiography showing the atrial septal defect rims A: TTE (4-chamber view) showing the atrio-ventricular rim B: TEE (bi-caval) view showing inferior and superior vena caval rims C: TEE (4-chamber) view showing posterior and atrioventricular rim D: TEE (short axis view) showing absence of aortic rim (red arrow) AoV: aortic valve; LA: left atrium; LV: left ventricle; RA: right atrium; RV: right ventricle; TEE: transesophageal echocardiography

**Figure 3:**
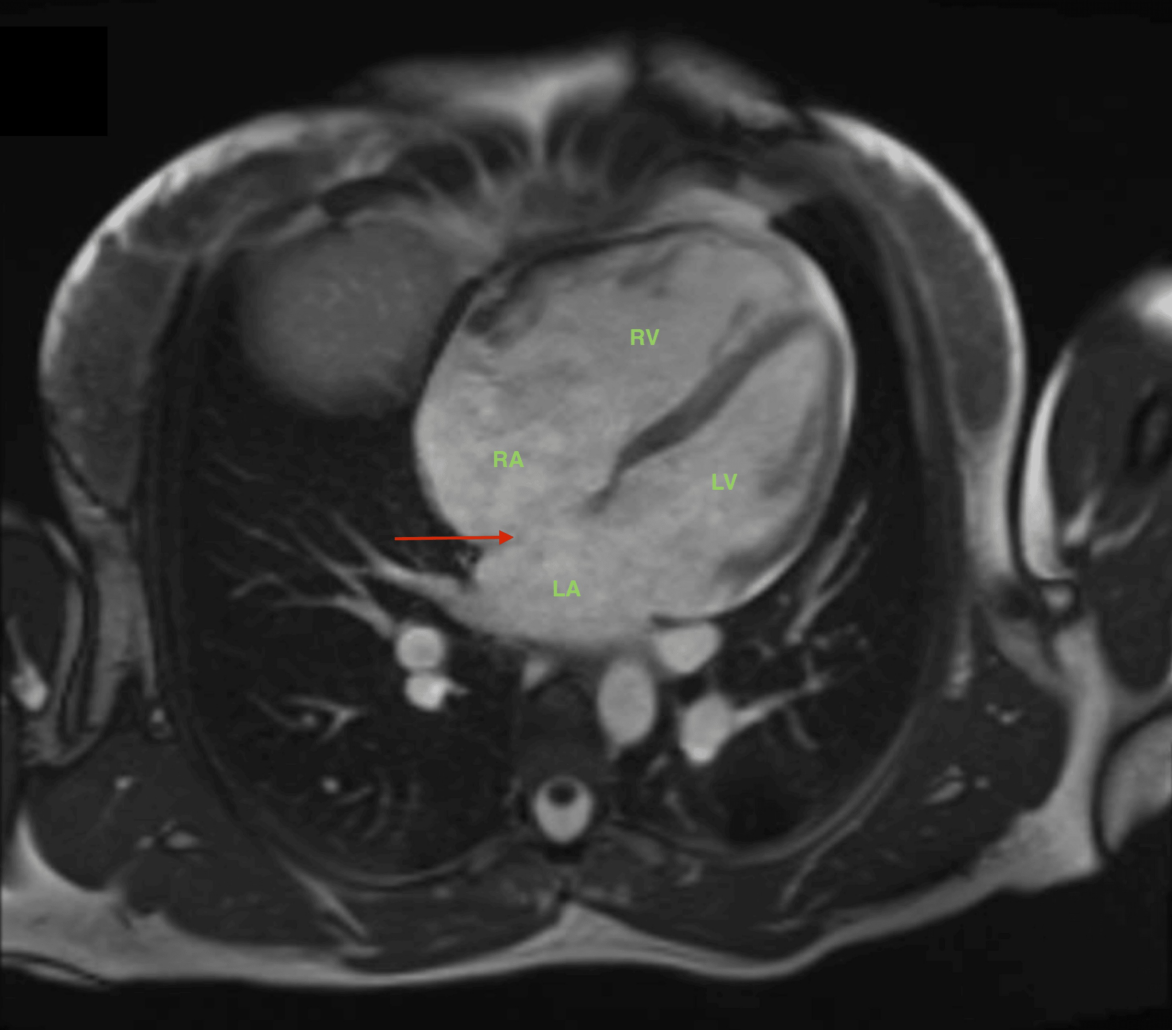
Preoperative cardiac MRI (transverse plane) demonstrating enlarged right cardiac cavities and a large atrial septal defect LA: left atrium; LV: left ventricle; RA: right atrium; RV: right ventricle Red arrow: atrial septal defect

Transthoracic echocardiography (Figure 2) revealed significant dilation of the right heart cavities associated with the presence of an inter-atrial defect. The patient had normal left ventricular ejection fraction and a measured cardiac output of 5l/min. This raised a suspicion of arrhythmogenic right ventricular cardiomyopathy and prompted a cardiac MRI for a more precise assessment. The cardiac MRI showed dilatation of the right cardiac cavities with a measured right ventricle end-diastolic volume of 295ml and end-systolic volume of 74ml, associated with the existence of large septal defect (Figure 3). Transesophageal echocardiography confirmed the presence of a large ostium secundum inter-atrial defect measuring 17mm x 13mm with a 3D surface of 1.8cm2, a dilatation of the right cardiac cavities and a calculated ratio between pulmonary and systemic blood flow (Qp/Qs) of 1.5. The ASD presented atrio-ventricular, superior vena caval, inferior vena caval and posterior rims but had an absent aortic rim (Figure 2).

The patient was admitted to the interventional cardiology department and a percutaneous closure procedure was performed under general anesthesia through right femoral venous access. Measurement was performed with a 22 x 19mm sizing balloon and a 24mm Amplatzer Septal Occluder was implanted. Intra-operative transoesophageal echocardiography showed a good positioning of the ASO with no residual shunt. There were no intra-operative complications. The patient was subsequently discharged from the hospital with a regimen of salicylic acid and clopidogrel.

**Figure 4:**
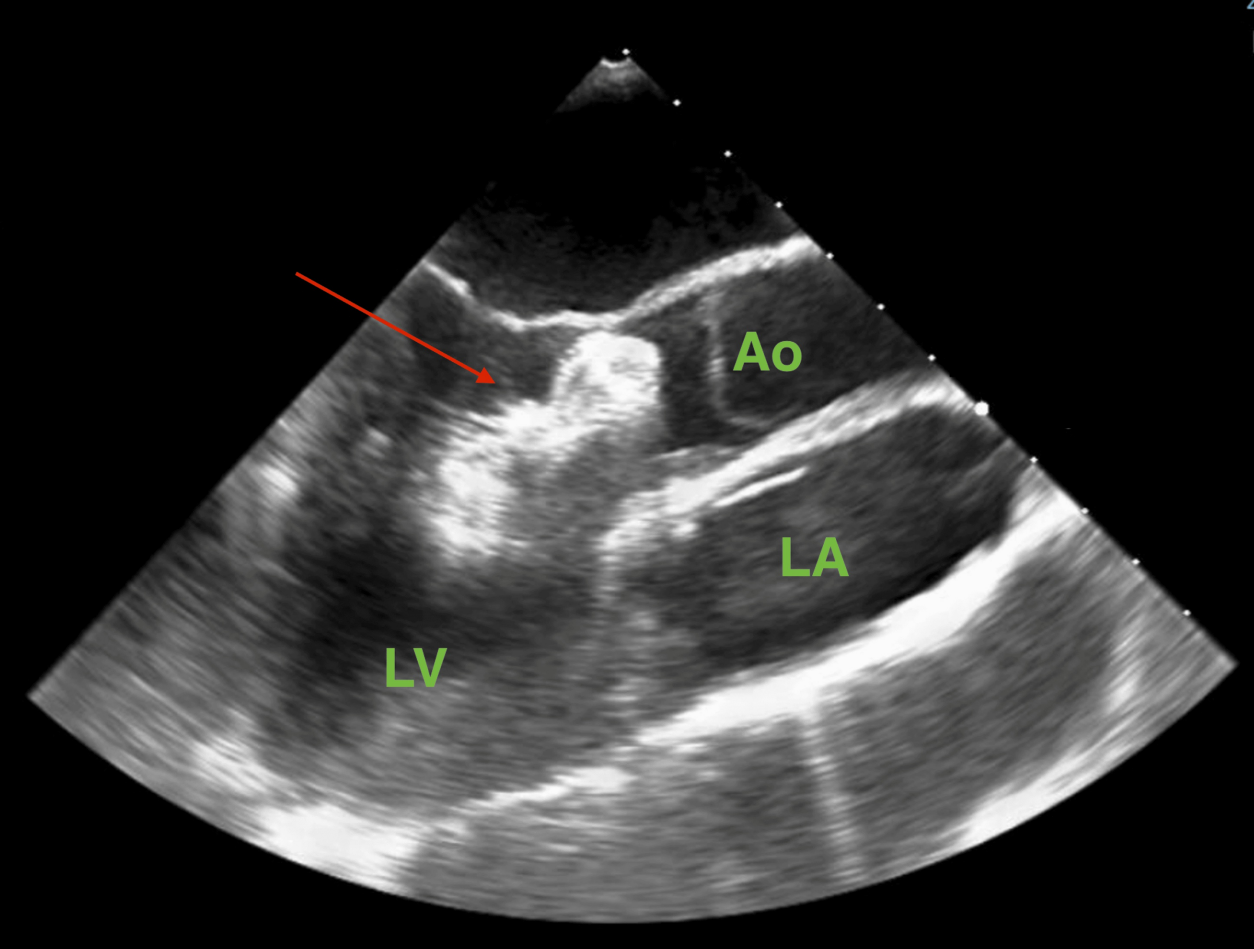
Echocardiography showing the atrial septal occluder trapped in the left ventricular outflow tract. Ao: aorta; LA: left atrium; LV: left ventricle Red arrow: Amplatzer Septal Occluder This echocardiography was performed seven weeks after atrial septal occluder implantation.

During a follow-up outpatient clinic visit, seven weeks after the percutaneous closure procedure, the patient still complained of palpitations. A control echocardiography revealed migration of the prosthetic device into the left ventricular outflow tract (Figure 4).

Considering the risk of embolisation to the aorta, a surgical procedure was immediately performed to extract the device and repair the inter-atrial defect. The surgical approach involved an inferior mini-sternotomy for access. Cardiopulmonary bypass was established with arterial and venous femoral cannulation, along with superior vena cava cannulation. A transthoracic aortic clamp was used. A right horizontal atriotomy provided access to the cardiac cavities allowing for the simple extraction of the prosthetic device through the ostium secundum. The defect was subsequently closed using a Supple Peri-Guard bovine pericardial patch. Cross-clamp time was 44min, total blood loss was 200cc. There were no intra-operative complications. The patient was discharged from the intensive care unit on day one and was ultimately discharged from the hospital on day six with a regimen of salicylic acid.

Twenty days after the surgical procedure, the patient presented at the emergency department complaining of palpitations. Chest CT showed a voluminous pleural effusion and EKG revealed atrial flutter. A pleural drainage was performed but medical treatment of atrial flutter with anti-arythmics was unsuccessful. Anticoagulation was initiated with Xarelto and the arrhythmia was subsequently treated by catheter ablation one month later.

**Figure 5:**
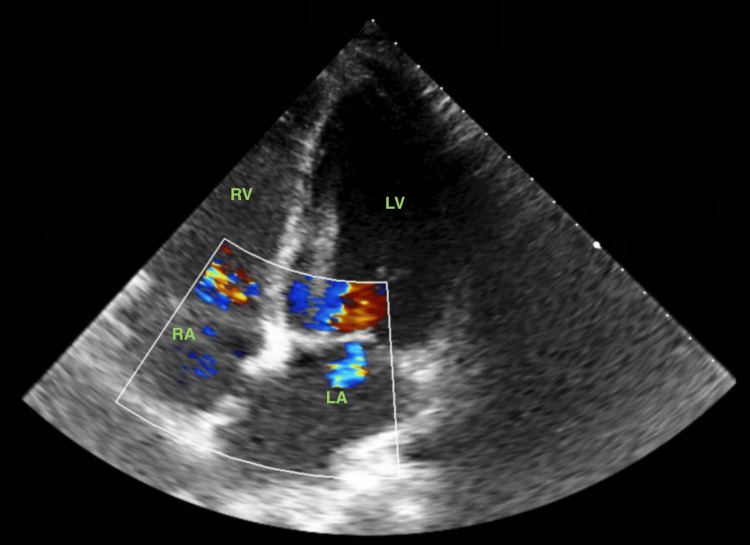
Doppler transthoracic echocardiography (4-chambers view) LA: left atrium; LV: left ventricle; RA: right atrium; RV: right ventricle This image was taken three months after surgical repair.

At outpatient clinic follow-up, at three-month post-operative, the echocardiography was unremarkable and showed no residual inter-atrial shunt (Figure 5). Chest radiography was clear and EKG showed sinusal rhythm and no arrhythmia, allowing for the discontinuation of anticoagulation.

## Discussion

Open surgical extraction was preferred to percutaneous retrieval for two reasons: firstly, due to the unstable position of the ASO in the left ventricular outflow tract and the subsequent risk of embolization into the aorta; and secondly, because of the uncertainty regarding factors that led to migration, the percutaneous deployment of a new ASO could be challenging and followed by subsequent new complications. The open approach allows for visual inspection and closure of all sizes and types of ASD regardless of the rims.

Pre-operative left-to-right shunt was moderate, prompting us to perform thorough assessments during reoperation to identify other factors that potentially led to device migration. The retrieved ASO presented no structural issue. There was no evidence of erosion into the atrium or aortic wall, a rare complication occurring in patients with deficient aortic rim and thought to be linked to undersized device or relative motion of the device to adjacent heart structures [11]. In our case, the patient had an absent aortic rim which led us to properly oversize the ASO [14]. Unlike inferior-posterior rim deficiency, isolated absence of the aortic rim does not represent a contraindication to ASO implantation and is not linked to a higher occurrence of major adverse events [7,15,16].

The cause and timing of migration remain uncertain, as the patient had no additional symptoms when diagnosed and no single definitive causative factor could be formally identified during reoperation. This underscores the importance of cautious implantation of ASO in patients with deficient aortic rim as well as vigilant post-operative follow-up to detect severe complications at an earlier stage.

## Conclusions

Careful patient selection, accurate sizing of the ASD and close follow-up are essential to ensure the success and safety of ASO implantation. Although oversizing the ASO can help minimize risks in case of deficient rims, it doesn’t fully prevent migration, making thorough preoperative assessment essential.

In this case, the exact cause and timing of migration were unclear, highlighting the importance of early monitoring. These findings emphasize the importance of a cautious and individualized approach, especially in patients presenting with a challenging anatomy like a deficient aortic rim.
